# The acute effects of whole blood donation on cardiorespiratory and haematological factors in exercise: A systematic review

**DOI:** 10.1371/journal.pone.0215346

**Published:** 2019-04-16

**Authors:** Diane Maria Johnson, Justin Roberts, Dan Gordon

**Affiliations:** Cambridge Centre for Sport & Exercise Sciences, School of Psychology & Sports Science, Anglia Ruskin University, Cambridge, United Kingdom; Belgian Red Cross-Flanders, BELGIUM

## Abstract

**Background:**

This systematic review aimed to collect the relevant historical and current literature to produce an informed analysis of the acute effects on cardiorespiratory and haematological factors following whole blood donation (~ 470 ml) during exercise. Testing the hypothesises that blood donation produces either no changes (Null) or produces significant changes (alternate) in haematology, $\dot{\text{V}}{\text{O}}_{2}$, heart rate, exercising power and time.

**Methods:**

Four databases of medical and science orientations were searched with terms sensitive to connections regarding exercise, blood donation (400–500 ml)/haematology, $\dot{\text{V}}{\text{O}}_{2}$, heart rate, exercising power and time. The study retrieval process utilised the PRISMA approach and selection was via an adapted scoring method according to the Consensus based Standards for the selection of health Measurements Instruments (COSMIN). Systematic review focused on 24–48 hrs post donation. Details of the PRSIMA checklist can be found in the accompanying online document.

**Results:**

Following scrutiny of 48 research papers by two independent assessors 8 experimental studies were included. Four studies showed a mean reduction for difference in $\dot{\text{V}}{\text{O}}_{2\text{max}}$ (- 2.4 ± 1.4 ml∙kg^-1^∙min^-1^) and a medium effect size (-0.26). No statistical significance was present at the mean meta-analysis level, also the case for heart rate, time to exhaustion and power. A mean reduction was seen in haemoglobin (- 1.05 g^.^dL^-1^), haematocrit (- 3.71%) and red blood cells (- 0.44 Mio μL^-1^), very large effect size was observed (Cohen’s *d*, -0.75, -1.16 and -4.23 respectively) and statistical significance (95% CI, -2.04, -0.54; -4.59, 2.28 and -4.37, -4.10 respectively).

**Conclusion:**

Although individual studies show that $\dot{\text{V}}{\text{O}}_{2\text{max}}$ Is reduced from blood donation pooled results show that $\dot{\text{V}}{\text{O}}_{2\text{max}}$ is indeed not significantly reduced from blood donation 24–48 hrs post donation. Additionally sub-maximally there isn’t enough data to produce substantial comparatives. Furthermore, this systematic review demonstrates that there are not enough high-quality studies regarding cardiorespiratory outcomes following blood donation.

## Introduction

Currently in the UK ~ 470 ml of blood is collected from donating a single unit [1] with blood transfusions distributed to treat medical conditions including cancer, anaemia and blood disorders, be used in surgery, including emergency and cardiac and for blood loss following child birth. In the UK alone 6,000 donations are needed every day and 200,000 new donors are needed each year in order to ensure that there is a sustained availability of blood [2].

A number of confounding reasons have been attributed to the progressive decline in new blood donors, including an increase in exotic holidays and tattoos [3,4] both of which can preclude the donation of blood coupled with an increasing population of people from non-white ethnic groups [5]. An additional population group who are potentially contributing to this decline are the sport enthusiasts and recreational/professional athletes, who may be concerned about the effect of BD on their performance [6]. Early concerns regarding BD affecting athletic performance were noted in the mid 1900’s where Springfield College, Massachusetts ruled that no man could be a blood donor while participating in varsity sport, due to reported unfavourable effects following BD such as wrestlers collapsing after competition and endurance lost in other athletes [7]. Additionally, reduced health, in terms of decreasing energy levels and becoming prone to infections or disease, as a result of BD is a fear of the non-donor [8]. Although not specific guidance for high-performance competitive athletes, the American Red Cross [9] suggest that a marginal decrease in exercise tolerance may be noticed up to a week after BD. Additionally, where exercise is concerned, according to NHS Blood and Transplant [10] and American Red Cross [9], prudence should be executed immediately after blood donation.

There are consequences to BD such as decrements in blood/plasma volume (B/PV) and haemoglobin (Hb). While the BV returns to normal within 24–48 hours (48–72 hrs, [11]), due to the progressive PV expansion [12], red cell mass and thus Hb restoration takes much longer, which according to Klein and Anstee [11] and NHS blood and Transplant guidelines [13] can be 3–6 weeks or 6–12 weeks respectively. Indeed, a restoration rate in Hb of 36 +/- 11 days was observed following removal of 550 ml of blood [14].

The biological responses to acute BD are well documented, indeed early work from Karpovich and Millman [7] highlighted that a Hb decrement affected athletic performance, while later work from Balke and colleagues [15] proposed that from a BD of 500 ml there was a 9% decline in maximal oxygen uptake ($\dot{\text{V}}{\text{O}}_{2\text{max}}$) and more recently Gordon and contemporaries [16] showed a 4.65% fall in $\dot{\text{V}}{\text{O}}_{2\text{max}}$ from a 450 ml donation. Additionally associated markers of aerobic fitness have been shown to be altered following BD such as maximum aerobic power output (W_max_) [17] and time to exhaustion (T_Ex_) [18,19,20]. There is little doubt that decreasing blood volume via BD will diminish oxygen carrying capacity, as haemoglobin (Hb) concentration will have been reduced and it is the primary source for the conveyance of oxygen. The iron containing protein Hb inside developing and mature red blood cells is important, in terms of arterial O_2_ content >99% of O_2_ is transported by Hb [21].

Currently the Cochrane Database regarding systematic review [22] shows no publications concerning the effects that BD has on exercise. There has however recently been a systematic review [23] concerning the effects of a standard whole blood donation on oxygen uptake and exercise capacity. The authors chose to include studies using a variety of blood volumes from 400–500 ml and across different donation time periods and exercise domains. The aim of this current systematic review is to build on the work from Van Remootel and colleagues [23] and produce an informed analysis of the acute effects of BD (~ 470 ml) on maximal oxygen uptake and associated cardio-metabolic markers (specific to haematology), within the time period of 12–24 hr post donation as it is suggested that exercise within the time period should be approached with caution [9,10]. Testing the hypothesis that blood donation produces either no changes or produces significant changes in haematology, $\dot{\text{V}}{\text{O}}_{2}$, heart rate (HR), exercising power and time.

## Method

The systematic review was carried out from guidance of Systematic Reviews [24] and by using the bibliographic management software RefWorks, Proquest, USA. Exact duplicates and close duplicates (a term that allows duplicates to be discovered despite minor differences being present, for example varying combinations of author names or differing titles and publication years), were assessed and then removed for subsequent analysis.

### Literature search of data base sources and search terms

From the beginning of December 2016 to the end of January 2018, Pubmed, Web of Science, SPORTDiscus and Scopus were used to obtain literature from the early 1900’s to January 2018, due to the databases being medical and science orientated. Search terms were created in order to be sensitive to all possible connections regarding exercise, blood donation/haematology, $\dot{\text{V}}{\text{O}}_{2}$, HR, exercising power and time. Hit numbers (which were absent from Van Remoortel et al. [23]) were Pubmed 3243, Web of Science 426, SPORTDiscus 249 and Scopus 5713. The following terms are an example of the search strategy used in Pubmed (a full strategy is available in the online supplementary material).

"blood donation” OR "blood collection" OR "blood withdrawal" OR "blood centre" OR "Blood center" OR "blood service" OR "blood bank" OR "blood transfusion" OR "phlebotom*" OR "plasma don*” OR “blood volume” OR “blood donor*”

"sport" OR "exercise" OR "physical endurance" OR "hiking" OR "cycling" OR "athletic" OR "training" OR "working out" OR "work out" OR "strenuous activity" OR “VO2max” OR “maximal aerobic power” OR “Oxygen carrying capacity”

“men” OR “man” OR “male” OR “sportsman” OR “sportsmen” OR “woman” OR “women” OR “female” OR “sportswomen” OR “Sportswoman” OR “athlete” OR “adult” OR “healthy” OR “humans” OR “people” OR “fit”

### Inclusion and exclusion criteria

Restricting the publications to English and using the Population, Interventions, Comparators, Outcomes and Study designs (PICOS) strategy enabled studies to be selected accordingly (see supplementary material). Studies that were accessible as conference abstracts were excluded, as were blood expansion studies, studies assessing muscular strength and studies involving participants > 66 years and < 17 years in age. The age range selected was according to blood transfusion services in the UK [25].

### Study retrieval process and quality assessment

The final studies selected for the systematic review were rated with an adapted scoring method [26] according to the Consensus based Standards for the selection of health Measurements Instruments (COSMIN) and the threshold for eligibility set at adequate or greater. For each sub-section of the tool a worst score counts scenario was adopted, with scores scaled from 4 (very good) to 1 (inadequate). This scoring method considers issues such as, control groups, allocation concealment, blinding, selective outcome reporting, quantity of cited publications, aims and hypothesis, appropriate variables, sampling method, size and description, validity and reliability of equipment, study design, descriptive statistics, statistics, research setting, calculations, inferences, findings and significances.

To ensure that the studies selected were of good quality, two assessors (DJ and DG) individually assessed the texts for eligibility according to the exclusion and inclusion criteria and additionally according to the previously mentioned COSMIN threshold.

### Data extraction and analysis

Using the spreadsheet software package Microsoft Excel, Microsoft Corp, Washington, USA (2011) a database was formed with the haematological and cardiorespiratory/respiratory variables extracted from the evaluated studies for analysis.

The variables of Hb, haematocrit (Hct), RBC’s, VO_2max_, maximum heart rate (MaxHR), maximum aerobic power output and time to exhaustion were all considered for inclusion in the systematic review, since they were the most widely reported variables relating to haematological and cardio-vascular responses and regarded according to their mean and standard deviation pre and post donation results. The post donation values were classified according to time frames, which included immediately, 2 hrs, 24–48 hrs, 48–72 hrs, 7 days, 14 days, 21 days and 28 days. However, for the purpose of this evaluation the systematic review focused on 24–48 hrs for two reasons. Firstly, the time period had sufficient data to analyse and secondly the time period is closest to being straight after donation and suggested by the NHS [10] and American Red Cross [9] to be approached with caution regarding exercise. For an outcome to be included for systematic review there needed to be at least two studies that reported a variable of interest. Valentine, Pigott and Rothstein, [27] suggest that although two studies is not ideal it will provide a conclusion and provides an analysis strategy.

Cohens *d* and 95% confidence intervals were calculated for each study. Cohens *d* (standardized difference in means) is expressed as the difference between two (raw) means divided by the pooled standard deviation (SD) of the groups (M1 –M_2_ / SD) [28]. Effect sizes for Cohens *d* are evaluated as, very large > 1.3, 0.8–1.3 (large), 0.5–0.8 (medium), < 0.2–0.5 (small). The mean Cohens *d* value was calculated by individual studies being weighted by the variance of the data, thus expressed as $\overset{-}{SE}=\frac{\Sigma ({ES}_{i}w_{i})}{\Sigma w_{i}}$ [29] or $Mean\;Cohens\;d=\frac{Sum(individual\;Cohen\;d\;from\;each\;study\;x\;inverse\;of\;variance\;from\;each\;study)}{Sum\;of\;inverse\;of\;variance}$.

## Results

**Fig 1:**
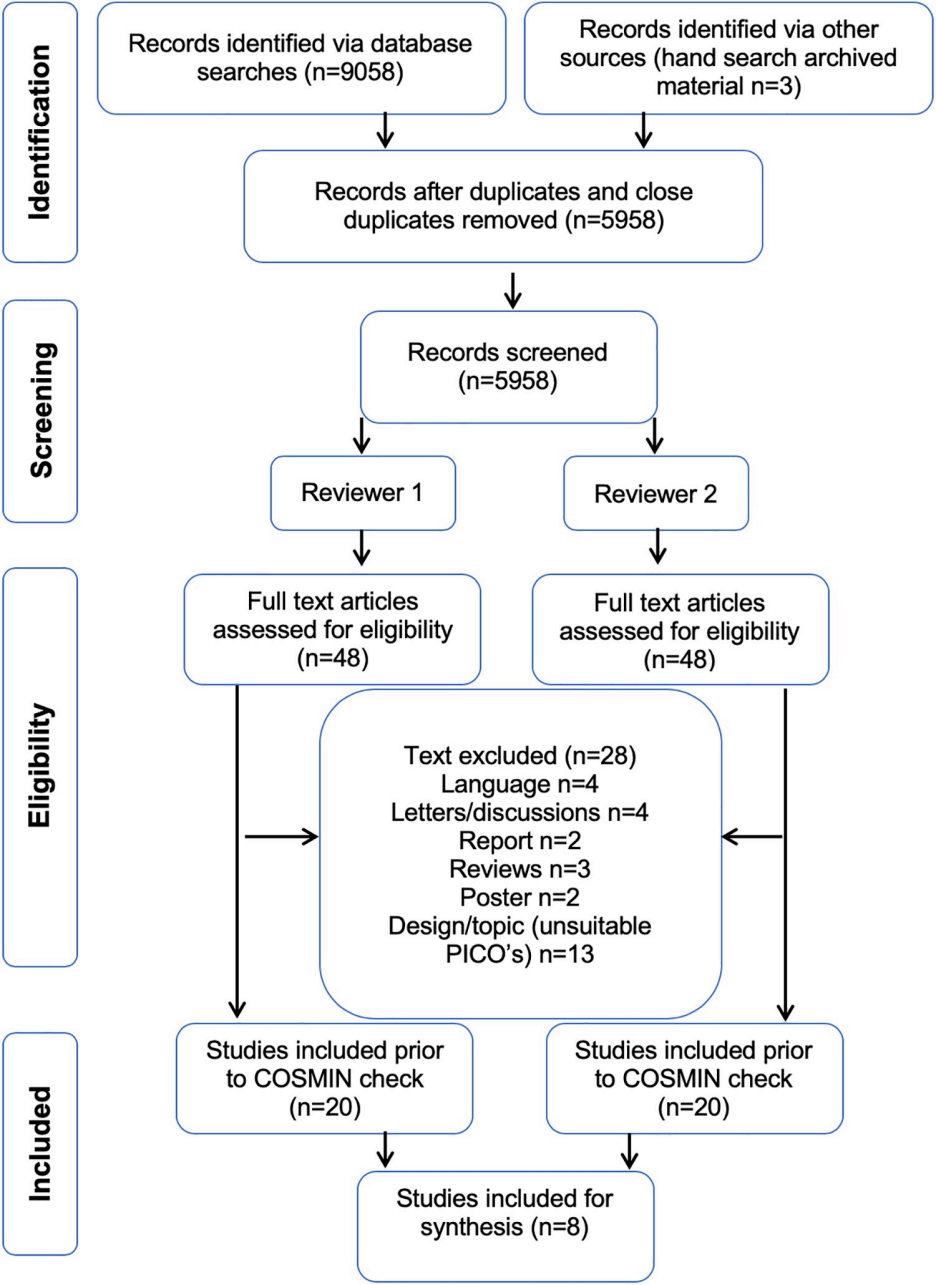
Flow diagram based on the PRISMA statement [30] illustrating the identification and selection process for the study retrieval.

Following scrutiny of 48 research papers for their eligibility and reducing that number to 20 after further inspection (regarding to PICO components), 8 studies were deemed suitable for inclusion in the systematic review following the COSMIN check, with the publication dates of those included ranging from 1997–2016 as highlighted in Fig 1. Regarding assessment of publication bias summarized effects (95% CI = -1.05,0.16 and *I*^2^ = 0%) of the main variable (VO_2max_) was assessed, and a Limits of Agreement plot showed that the two assessors were considered to be in agreement (bias = 0.12, CI = -0.25,0.55.

### Overview of studies included

All 8 studies included were experimental in nature, incorporating a before and after blood donation approach, with only the Meurrens, et al. [20] study using a control (sham bleed) group. Participant numbers ranged from 9–24 (13.5 ± 4.9) and hence appeared low, unfortunately only two studies, that of Judd, et al. [31] and Meurrens, et al. [20] provided power calculations to reflect sample size, although justification for sample size was presented in all studies except Krip, et al. [32].

The majority of the participants were male (91%) and the mean age of all who took part was 23.9 ± 1.5 with a mean height of 179.2 ± 3.4 cm and a mean mass of 76.4 ± 5.9 Kg.

### Cardiorespiratory variables

#### Oxygen Uptake ($\dot{\text{V}}{\text{O}}_{2}$)

The exercise mode for all studies was cycle ergometer, with a protocol that induced exhaustion and thus $\dot{\text{V}}{\text{O}}_{2\text{max}/\text{peak}}$ with the exception of Gordon, et al. [33] and Krip, et al. [32], which were submaximal studies. Table 1 shows an overview of the protocols used by all researchers.

**Table 1:** Overview of the 9 studies included. Sample size varied, as did the combination of male (M) and female (F) participants. Blood donation volumes ranged from 450–500 ml. With age, height and mass reported as mean ± SD.

Study	Sample, size (N) & sex (M/F)	Blood drawn (ml)	Age (yrs)	Height (cm)	Mass (Kg)	Exercise mode & overview of protocol
Burnley, et al., 2006	10 (M) 1 (F) Physically active & healthy	450	23 ± 6	177 ± 4.0	77.2 ± 11	Cycle: 0 W for 3 min then 80% of difference between GET and $\dot{\text{V}}{\text{O}}_{2\text{peak}}$ (severe intensity domain) to exhaustion.
Gordon, et al., 2010	6 (M) 4 (F) Regular sport participants	450	21 ± 2	175.2 ± 5.1	66.4 ± 2.8	Cycle: Test 1, 6 min at 80% of respiratory exchange ratio or Test 2, 50% of the difference between $\dot{\text{V}}{\text{O}}_{2\text{max}}$ and respiratory exchange ratio.
Gordon, et al., 2013	15 (M) Well trained athletes	450	23.3 ± 4.5	180.1 ± 6	77.4 ± 13.1	Cycle: increase ramp from 100W by 25w/min at 80rpm to exhaustion.
Hill, Vingren & Burdette., 2013	2 (F), 7 (M) Healthy uni students	450	23 ± 1 (F) 25 ± 3 (M)	170 ± 11 (F) 182 ± 2 (M)	60 ± 8 (F) 92 ± 15 (M)	Cycle: Following a 4 min warm up at 79 ± 15 W and 4 min rest, 80rpm held at 274 ± 2 W to exhaustion.
Judd, et al., 2011	2 (F) 10 (M) Moderately active	450	24.3 ± 5.2	181 ± 8	87.5 ± 15.3	Cycle: starting at 1.5 /1.0 kp (men and women respectively), ramp increase of 0.5 kp per 2min at 80rpm to exhaustion.
Krip, et al., 1997	12 (M) (6 gave blood) Endurance trained	500	24.8 ± 0.5	N.D.	73.9 ± 2.2	Cycle: 6 min at 60rpm at successive work rates to attain target HR’s (maximum of 180).
Meurrens, et al., 2016 ^#^	24 (M) (16 gave blood) Moderately trained	470	25.6 ± 0.76	180.2 ± 1.82	73.8 ± 1.96	Cycle: Starting at 70W and increasing by 30W every 2 min at 85-90rpm to exhaustion.
Ziegler, et al., 2014	20 (M) Healthy men	450	25.9 ± 1.6	185 ± 2	78.7 ± 3.3	Cycle: 6 min warm up at 100W, 35W/min to exhaustion.

N.B. N.D. denotes no data,

^#^denotes this study is a RCT design and as such is not included in the meta-analysis.

Gordon, et al. [33] has submaximal data in the moderate (below Gas Exchange Threshold (GET), specifically 80% of the difference between rest and GET) and heavy domains (between GET and $\dot{\text{V}}{\text{O}}_{2\text{max}}$, which was 50% of the difference between GET and $\dot{\text{V}}{\text{O}}_{2\text{max}}$), but cannot be compared to any other studies due to no parallels. Similarly, with little data to compare against and no apparent parallels this is the case also for the Krip, et al. [32] study.

The participants athletic descriptions (Table 1) included physically active and healthy to well-trained athletes, resulting in $\dot{\text{V}}{\text{O}}_{2\text{max}}$ reported mean value before blood donation of 51.31 ± 7.10 ml∙kg^-1^∙min^-1^ (Table 2) (when converting all the values (including Burnley and team [18] and to ml∙kg^-1^∙min^-1^). With values ranging from 40 ± 4.0 [19] to 64.1 ± 1.9 ml∙kg^-1^∙min^-1^ [32].

**Table 2:** $\dot{\text{V}}{\text{O}}_{2\text{max}}$ values pre donation (Unbled) in all 8 studies are shown with post donation (Bled) values for the studies that collected 24–48 hrs data. Gordon, et al. [33] was a submaximal test post donation in the moderate and heavy domains involving oxygen uptake kinetics and thus the values are not reported in this table.

Study	$\dot{\text{V}}{\text{O}}_{2}$ & $\dot{\text{V}}{\text{O}}_{2\text{max}/\text{peak}}$ ml∙kg^-1^∙min^-1^ (unless otherwise specified)	
	Unbled	Bled (24-48hrs)
Burnley, et al., 2006	3.79 ± 0.64 *^♯^	3.64 ± 0.61 *^♯^
Gordon, et al., 2010	53.0 ± 4.1	-
Gordon, et al., 2013	51.3 ± 7.6	48.4 ± 7.9 ^
Hill, Vingren & Burdette., 2013	40 ± 4.0	36.0 ± 4.0
Judd, et al., 2011	46.6 ± 7.0	44 ± 6.5
Krip, et al., 1997	64.1 ± 1.9 ^$^	-
Meurrens, et al., 2016	56.7 ± 1.5	53.9 ± 1.4
Ziegler, et al., 2014 [34]	49.7 ± 1.7^♯^	-

N.B.

* denotes values in l∙min^-1^,

^ denotes that this time period was 48-72hrs.

^♯^ denotes the Burnley [18] study post donation showing a $\dot{\text{V}}{\text{O}}_{2\text{peak}}$ value, as this was a test performed in the severe intensity domain to exhaustion and the Ziegler, et al. study showing $\dot{\text{V}}{\text{O}}_{2\text{peak}}$ values, with their criteria to exhaustion and a respiratory exchange ratio of more than 1.2.

^$^ denotes the Krip, et al. [32] study showing submaximal unbled end $\dot{\text{V}}{\text{O}}_{2}$ values.

**Fig 2:**
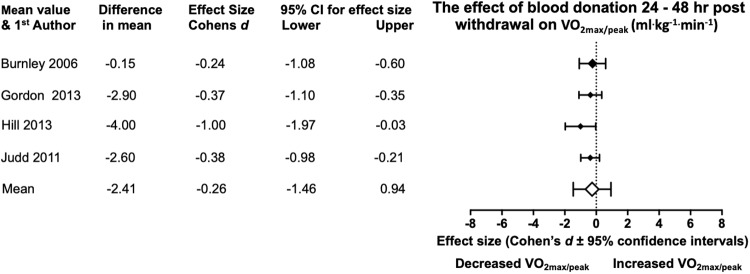
Statistical results from four studies of pre and 24–48 hr post BD in $\dot{\text{V}}{\text{O}}_{2\text{max}/\text{peak}}$. With the diamonds reflecting the effect size of the studies. The white diamond shows the overall mean effect size. All effect sizes are Cohen’s *d* and 95% confidence interval, with heterogeneity of *I*^2^ = 0%.

Table 2 shows that regardless of the time frame in a bled situation there is a trend for BD to decrease $\dot{\text{V}}{\text{O}}_{2\text{max}}$ values from the pre donation values. With focus on the time frame of 24–48 hrs post donation, under meta-analysis examination, evidence from the four studies within this bled time period showed a mean reduction for difference in $\dot{\text{V}}{\text{O}}_{2\text{max}}$ (- 2.4 ± 1.4 ml∙kg^-1^∙min-1), with a small effect size, -0.26 (Fig 2). No statistical significance is present at the mean meta-analysis level due to the 95% CI passing through 0 (-1.46, 0.94). Hill, Vingren & Burdette [19] had significance in the decreased of VO_2max_ with a large effect size (Cohen’s *d*, -1) and confidence intervals of -1.97 to -0.03. Of the remaining studies all had small Cohen’s *d* effect sizes, but no statistical significance.

#### Heart rate (HR)

**Fig 3:**
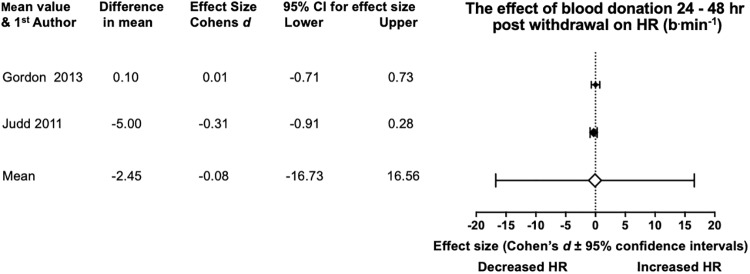
Statistical results from two studies of pre and 24–48 hr post BD in HRmax. With the diamonds reflecting the effect size of the studies. The white diamond shows the overall mean effect size. All effect sizes are Cohen’s *d* and 95% confidence interval, with heterogeneity of *I*^2^ = 0%.

The HR pre and post donation values were only acknowledged in three studies (Table 3), with Krip, et al. [32] only presenting post donation HR results. On viewing, the values are similar across time frames. Indeed, the meta-analysis showed no statistical significance for any of the results, all the CI’s passed through “0”, with very small Cohens *d* effects across all research too (Fig 3).

**Table 3:** Maximal HR values pre donation (Unbled) and post donation (Bled) over various time frames.

Study	HRmax (b∙min^-1^)	
	UnBled	Bled (24-48hrs)
Gordon, et al., 2013	184.9 ± 10.1	185.0 ± 10 ^
Judd, et al., 2011	186 ± 12	181 ± 19
Krip, et al., 1997	N/A	187 ± 3
Meurrens, et al., 2016	187±4	187 ± 3

N.B.

^ denotes that this time period was 48-72hrs. No pre value was available for Krip, et al. [32]

#### Time to exhaustion (TEx)

**Fig 4:**
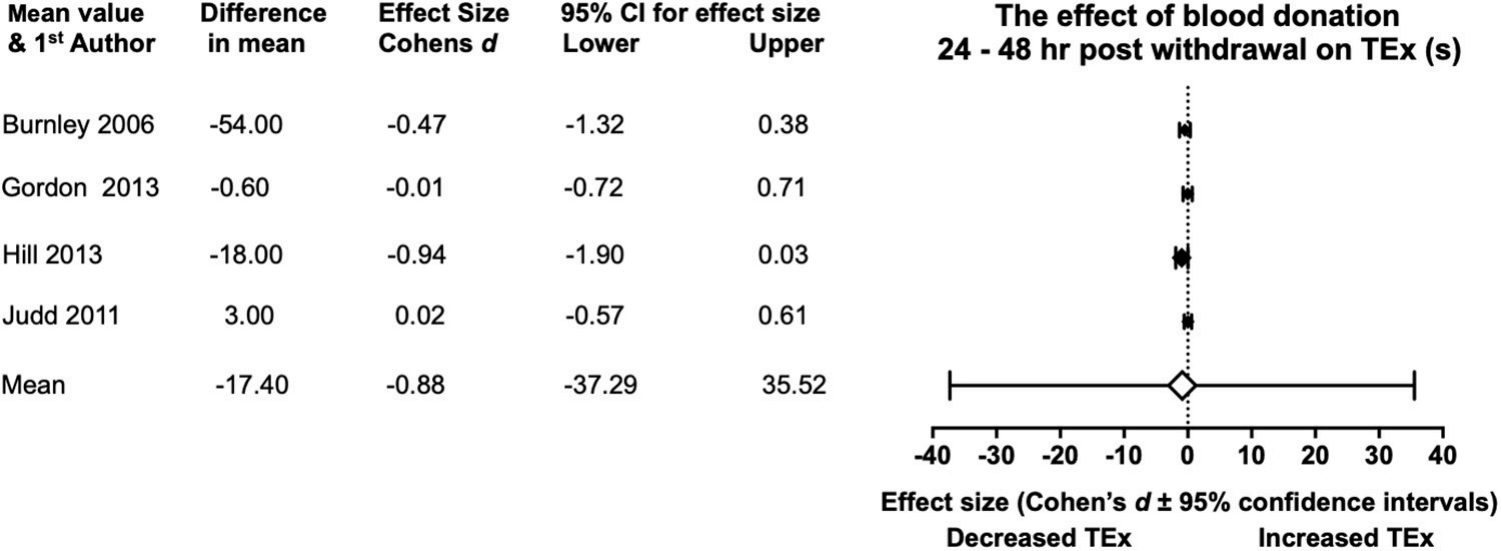
Statistical results from four studies of pre and 24–48 hr post BD in TEx (s). With the diamonds reflecting the effect size of the studies. The white diamond shows the overall mean effect size. All effect sizes are Cohen’s *d* and 95% confidence interval, with heterogeneity of *I*^2^ = 11.57%.

TEx pre and post donation evidence was recorded from five studies (Table 4), which are the same groups shown in Table 2, which related to $\dot{\text{V}}{\text{O}}_{2\text{max}/\text{peak}}$ results. With the exception of the Judd and contemporaries [31] study TEx shows an inclination to decrease in a Bled condition from Unbled across all time frames. For the time frame of 24–48 hr the meta-analysis (Fig 4) showed a mean reduction in TEx (- 17.40 s), with a large effect size produced (Cohen’s *d*, -0.88). No statistical significance is present at the mean meta-analysis level due to the 95% CI passing through 0 (-37.29, 35.52).

**Table 4:** TEx pre and post donation (Unbled and bled) values over various time periods.

Study	TEx (s)	
	UnBled	Bled (24-48hrs)
Burnley, et al., 2006	375 ± 129	321 ± 99
Gordon, et al., 2013	611.4 ± 101.4	610.8 ± 109.8 ^
Hill, Vingren & Burdette., 2013	268 ± 16	250 ± 22
Judd, et al., 2011	586 ± 168	589 ± 189
Meurrens, et al., 2016	1014 ± 54	966 ± 60

N.B.

^ denotes that this time period was 48-72hrs.

#### Maximum aerobic power output (W_max_)

W_max_ pre (329.7 ± 42.4) and post (329.5 ± 45.7) donation evidence could only be gathered from one study [16] and the time period for this was 48-72hrs, thus no meta-analysis could be performed.

### Haematological variables

#### Hb

**Fig 5:**
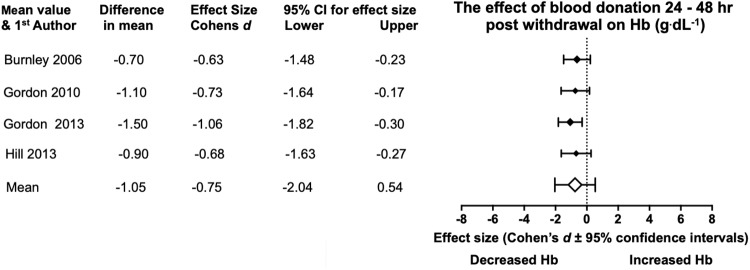
Statistical results from four studies of pre and 24–48 hr post BD in Hb. With the diamonds reflecting the effect size of the studies. The white diamond shows the overall mean effect size. All effect sizes are Cohen’s *d* and 95% confidence interval, with heterogeneity of *I*^2^ = 0%.

Hb pre and post donation evidence was recorded from five studies (Table 5), across these studies there is a decrement in Hb under Bled conditions, which reveals an 8.06% decrease in Hb levels. The meta-analysis (Fig 5) showed a mean reduction in Hb (- 1.05 g^.^dL^-1^) across four relevant studies, with a medium effect size produced (Cohen’s *d*, -0.75). No statistical significance is present at the mean meta-analysis level due to the 95% CI passing through 0 (-2.04, 0.54). Gordon, et al. [16] showed a large effect size and statistical significance as confidence intervals were to the left of 0 (1.82, -0.30). Cohen’s *d* effects were moderate to high across all the studies. Burnley, et al. [18], Gordon, et al. [33] and Hill, et al. [19] were not shown to have statistical significance under meta-analysis scrutiny for Hb.

**Table 5:** Hb pre and post donation (Unbled and bled) values over various time frames, with the Krip, et al. [3,2] and Ziegler, et al. [3,4] studies not reporting post donation values.

Study	Hb (g^.^dL^-1^)	
	UnBled	Bled (24-48hrs)
Burnley, et al., 2006	15.4 ±0.9	14.7 ± 1.3
Gordon, et al., 2010	14.2 ± 1.5	13.1 ± 1.5
Gordon, et al., 2013	15.6 ±1.2	14.1 ± 1.6 ^
Hill, Vingren & Burdette., 2013	14.9 ± 0.8	14.0 ± 1.7
Krip, et al., 1997	15.8 ± 0.5	-
Meurrens, et al., 2016	15.6 ± 0.2	13.7 ± 0.3
Ziegler, et al., 2014	9.3 ± 0.11 *	-

N.B.

* denotes values in mmol/L and

^ denotes that this time period was 48-72hrs.

#### Hct

**Fig 6:**
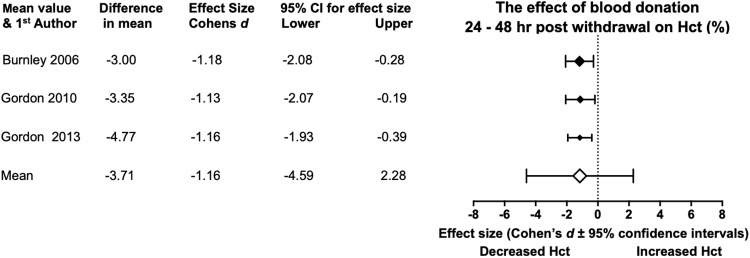
Statistical results from three studies of pre and 24–48 hr post BD in Hct. With the diamonds reflecting the effect size of the studies. The white diamond shows the overall mean effect size. All effect sizes are Cohen’s *d* and 95% confidence interval, with heterogeneity of *I*^2^ = 0%.

Hct pre and post donation evidence was recorded from four studies (Table 6). Across these studies there is a tendency for Hct to decline in a Bled state, revealing an 8.81% decrease in Hct levels. From Unbled to Bled the meta-analysis (Fig 6) across the three relevant studies [16,18,33] showed a mean reduction in Hct (-3.71%) and a large mean effect size (Cohen’s *d*, -1.16). Statistical significance is not present at the mean meta-analysis level due to the 95% CI passing through 0 (-4.59, 2.28). All studies show large effect sizes and statistical significance.

**Table 6:** Hct pre and post donation (Unbled and bled) values over varying time frames.

Study	Hct (%)	
	UnBled	Bled (24-48hrs)
Burnley, et al., 2006	44 ± 2	41 ± 3
Gordon, et al., 2010	43.55 ± 3.28	40.20 ± 2.63
Gordon, et al., 2013	48.76 ± 4.31	43.99 ± 3.91 ^
Krip, et al., 1997	41.1 ± 1.2	-
Meurrens, et al., 2016	45.8 ± 0.6	40.9 ± 0.9
Ziegler, et al., 2014	43.7 ± 0.6	-

N.B.

^ denotes that this time period was 48-72hrs.

#### RBC’s

**Fig 7:**
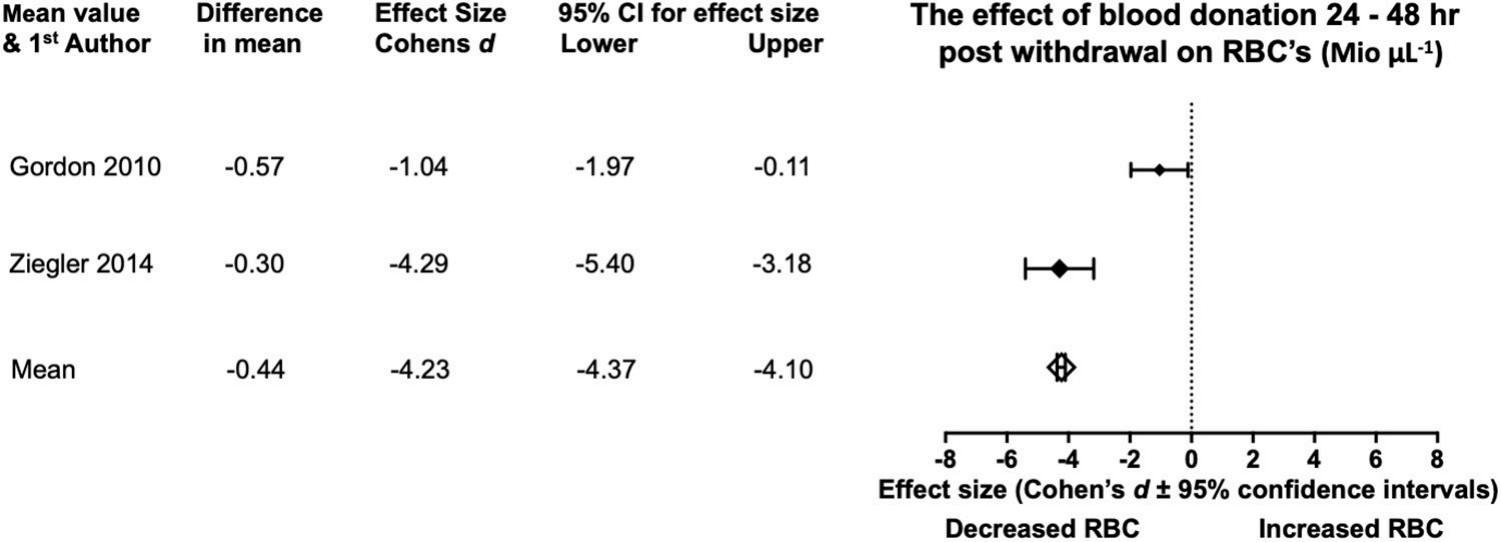
Statistical results from two studies of pre and 24–48 hr post BD in RBC’s. With the diamonds reflecting the effect size of the studies. The white diamond shows the overall mean effect size. All effect sizes are Cohen’s *d* and 95% confidence interval, with heterogeneity of *I*^2^ = 0%.

RBC’s pre and post donation evidence was recorded from three studies (Table 7). Across all studies there is a tendency for RBC’s to decline in a Bled state this reveals a 9.49% decrease in RBC’s levels. From Unlbed to Bled the meta-analysis (Fig 7) across the two relevant studies [16,34] revealed a mean reduction in RBC’s (-0.44 Mio μL^-1^) and a very large mean effect size (Cohen’s *d*, -4.23). Statistical significance is present at the mean meta-analysis level due to the 95% CI being placed notably to the left of 0 (-4.37, -4.10). All studies show large to very large effect sizes and statistical significance.

**Table 7:** RBC’s pre and post donation (Unbled and bled) values over varying time frames.

Study	RBC's (Mio μL^-1^)	
	UnBled	Bled (24-48hrs)
Gordon, et al., 2013	5.28 ± 0.50	4.71 ± 0.59 ^
Meurrens, et al., 2016	5.2 ± 0.1	4.6 ± 0.1
Ziegler, et al., 2014	4.9 ± 0.07	4.6 ± 0.07

N.B.

^ denotes that this time period was 48-72hrs.

## Discussion

Based on the hypothesis stated that blood donation produces either no changes (H_0_) or produces significant changes (H_1_) in haematology, $\dot{\text{V}}{\text{O}}_{2}$, heart rate (HR), exercising power and time, the following can be concluded. $\dot{\text{V}}{\text{O}}_{2\text{max}/\text{peak}}$ based on the mean Cohen’s *d* ± 95% confidence intervals appears to accept the Null hypothesis, as the line passes through “0” and thus is not statistically significant; this is also the case for HR, TEx and W_max_. For the blood variables the alternate hypothesis is accepted only for RBC’s, rejecting the Null hypothesis, as the mean Cohen’s *d* ± 95% confidence intervals favour donation.

Research papers have historically determined that BD decreases $\dot{\text{V}}{\text{O}}_{2\text{max}}$ [15,16,19,20] However, in this analysis the confidence intervals have not produced statistical significance. Individual studies have too much variance and therefore the pooled data using the Cohen’s *d* allows for comparisons across all papers. A potential confounding reason for the lack of significance could be the absence of statistical power due to low sample sizes. Indeed, this systematic review had the potential to include 20 papers, but due to quality issues, only 9 papers were considered to be of good quality, none of the papers were considered to be excellent quality, based on the adapted scoring method [26]. Reasons for exclusion were heterogeneous, but a reoccurring theme even in the good quality papers was lack of a power calculation, indeed only the Meurrens, et al. [20] and Judd, et al. [31] studies that were in the list examined provided this.

An overall mean reflection of blood variables for the systematic review data collected showed that under Bled conditions (24–48 hr) RBC’s, Hct and Hb values were significantly reduced. Where data allowed there were three studies [16,18,32] in this systematic review that showed proportional reductions in Hct and Hb post donation. Mathematically for normal haematocrit (37–41/43% and 42–47% in women and men respectively [35,36]) of 15g Hb/100ml (blood), the Hb carrying capacity of O_2_ = 1.34 x 15 = 20.1 mL O_2_/100mL [37]. This would equate to loosing 94.47 mL O_2_ and 70.5 g Hb in a 470 ml blood donation, which has been postulated to affect variables such as $\dot{\text{V}}{\text{O}}_{2\text{max}}$ decreasing [16,19,20]. Therefore, it seems prudent for all studies that involve blood donation and exercise together to collect relevant haematological variables, such as Hb, Hct, RBC’s. Yet, various authors neglected to report these values fully.

One issue worth noting is that only one study [16] controlled for hydration adequately, in the other studies it was not controlled properly or sometimes not mentioned at all. It is important as; dehydration (or even over hydration) affects blood-based readings. Hct and Hb will alter according to hydration levels. It is thus suggested that for maintaining hydration (in a clinical setting) fluid intake should be 35 ml.kg^-1^ for 18–60 yrs and 30 ml.kg^-1^ for over 60 yrs [38]. Therefore, the variance in fluids could be attributed to the change in fat-free mass and adiposity that can alter with age, as fat-free mass is ~ 70–80% water [39] and muscle mass diminishes with age [40] particularly if there is inactivity.

Although in many of these studies Hb, Hct and RBC’s were the considered blood variables only two studies [20,34] considered the iron storage protein, ferritin concentration. Approximately 25% of the body’s iron is stored as ferritin (NHS, 2017) and repeated blood donations decrease its levels, as shown in the Meurrens, et al. [20] study where 3 blood donations (taken 3 months apart from one another) were observed. The results saw ferritin prior to any donation at 55 ± 8 μg/l to then reduce to 40 ± 6 μg/l (*p* < 0.01) before the second donation, and a value of 41 ± 5 μg/l (*p* < 0.001) was observed prior to the third donation, It is not known in some of the studies if the participants are new or regular donors, iron status according to ferritin levels could aid in indicating normal levels (41–400 μg/l and borderline 16–40 μg/l (NHS, 2017)). This might be important to help provide insight on differing results between studies.

Further consideration when comparing studies should be given to the heterogeneous mix of participants, for example a study declares the participants to be trained athletes [16], but fails to mention what their training history was, while another study reported to have physically active and healthy participants [18], but no quantification of this was provided. Quantification could come in the form of analysing individual $\dot{\text{V}}{\text{O}}_{2\text{max}}$ results, for example by using ACSM [41] guidelines and observing the combined values for Gordon and colleagues [16] study reveals the participants were of excellent fitness (in terms of aerobic power) and the participants for Burnley [18] and associates were of good fitness. Thus, greater transparency is required and authors should provide this type of information.

The only study in this systematic review to use a randomized controlled trial (sham bleed) was that of Meurrens and colleagues [20] and hence this study was not pooled into the relevant forest plots and subject of the meta-analysis. Thus, it can be thought with confidence that measures to reduce bias were adopted to test the effectiveness of the intervention (blood donation) in this study, while the other studies involved opted not to operate in this manner. The Meurrens [20] group blinded the participants and they all went through the process of donation, however some were not aware that they had not donated blood (sham). The positive results that are associated with this type of approach should be appreciated. Blinding helps to ensure impartiality and that bias is eliminated or at least reduced.

Further analysis showed that with the exception of HR, Meurrens and associates [20] would have had clear statistical significance across all variables and very large effect sizes with for example a Cohen’s *d*, -1.93 for VO_2max_ and -6.01 for Hb., if they were included in the meta-analysis.

However, the pooling of robust high quality randomized controlled studies with lower quality uncontrolled designs is not recommended and would possibly lead to type I errors.

## Conclusion

In summary, it is clear from this systematic review that there are not enough high-quality papers to draw any definitive conclusions, regarding cardiorespiratory outcomes following BD. Although we have an understanding from individual study results of the raw data that $\dot{\text{V}}{\text{O}}_{2\text{max}}$ is reduced from donation, pooled results show that $\dot{\text{V}}{\text{O}}_{2\text{max}}$ is indeed not significantly reduced from blood donation 24–48 hrs post donation. Additionally sub-maximally data is limited for substantial comparatives. Moreover, there is a need for further exploration regarding post blood donation health and well-being in athletes and non-athletes alike. Generally the papers evaluated in this instance portrayed the participants as generally active/athletic and additionally they were all young, but this does not necessarily reflect the general public (who donate blood), thus this avenue needs to be investigated further encompassing a more reflective cohort of the people. To conclude, pending superior quality studies the current evidence cannot provide definitive answers regarding cardiorespiratory variables, but it can report the reductions in the blood variables investigated, which potentially impact sporting ability.

## Supporting information

S1 Table Search terms and hits table. (DOCX)

S2 Table PICO table. (DOCX)

S3 Table Variables extracted for analysis. (DOCX)

S4 Table Excluded papers information. (DOCX)

S5 Table PRISMA checklist. (DOC)
