# Supplementary material for: The acute effects of whole blood donation on cardiorespiratory and haematological factors in exercise: A systematic review
Source: PLoS One. 2019 Apr 16;14(4):e0215346. doi: 10.1371/journal.pone.0215346 (PMC6467450; doi:10.1371/journal.pone.0215346)
Supplement: S1 Table — (DOCX) [file pone.0215346.s001.docx]

| **Data Base** | **Hits** | **Search Terms** |
| --- | --- | --- |
| Scopus | 5713 | "blood donation” OR "blood collection" OR "blood withdrawal" OR "blood centre" OR "Blood center" OR "blood service" OR "blood bank" OR "blood transfusion" OR "phlebotom*" OR "plasma don*” OR “blood volume” OR “blood donor*”  "sport" OR "exercise" OR "physical endurance" OR "hiking" OR "cycling" OR "athletic" OR "training" OR "working out" OR "work out" OR "strenuous activity" OR “VO2 max” OR “maximal aerobic power” OR “Oxygen carrying capacity”  “men” OR “man” OR “male” OR “sportsman” OR “sportsmen” OR “woman” OR “women” OR “female” OR “sportswomen” OR “Sportswoman” OR “athlete” OR “adult” OR “healthy” OR “humans” OR “people” OR “fit” |
| Web of Science | 426 | (TS=("blood don*") OR TS=("blood collection") OR TS=(“blood withdrawal") OR TS=("blood centre") OR TS=("Blood center") OR TS=(“blood service") OR TS=("blood bank") OR TS=("blood transfusion") OR TS=("phlebotom*") OR TS=("plasma don*”)) AND (TS=("sport*") OR TS=("exercis*") OR TS=("physical endurance") OR TS=("hiking") OR TS=("cycling") OR TS=("athletic") OR TS=("training") OR TS=("working out") OR TS=("work out") OR TS=("strenuous activity") OR TS=(“VO2max”) OR TS=(“maximal aerobic power”) OR TS=(“VO2 max”) OR TS=(“Oxygen carrying capacity”)) AND (TS=(“men”) OR TS=(“man”) OR TS=(“male”) OR TS=(“sportsman”) OR TS=(“sportsmen”) OR TS=(“woman”) OR TS=(“women”) OR TS=(“female”) OR TS=(“sportswomen”) OR TS=(“Sportswoman”) OR TS=(“athlete”) OR TS=(“adult”) OR TS=(“healthy”) OR TS=(“humans”) OR TS=(“people”) OR TS=(“fit”)) |
| Pub-med | [3243](https://www.ncbi.nlm.nih.gov/pubmed) | "blood donation” OR "blood collection" OR "blood withdrawal" OR "blood centre" OR "Blood center" OR "blood service" OR "blood bank" OR "blood transfusion" OR "phlebotom*" OR "plasma don*” OR “blood volume” OR “blood donor*”  "sport" OR "exercise" OR "physical endurance" OR "hiking" OR "cycling" OR "athletic" OR "training" OR "working out" OR "work out" OR "strenuous activity" OR “VO2 max” OR “maximal aerobic power” OR “Oxygen carrying capacity”  “men” OR “man” OR “male” OR “sportsman” OR “sportsmen” OR “woman” OR “women” OR “female” OR “sportswomen” OR “Sportswoman” OR “athlete” OR “adult” OR “healthy” OR “humans” OR “people” OR “fit” |
| SPORTDiscus | 249 | TI "blood don*" OR TI "blood collection" OR TI “blood withdrawal" OR TI "blood centre" OR TI "Blood center" OR TI “blood service” OR TI "blood bank" OR TI "blood transfusion" OR TI "phlebotom*" OR TI "plasma don*” OR AB "blood don*" OR AB "blood collection" OR AB “blood withdrawal" OR AB "blood centre" OR AB "Blood center" OR AB “blood service” OR AB "blood bank" OR AB "blood transfusion" OR AB "phlebotom*" OR AB "plasma don*” OR TI “blood volume” OR TI “blood donor*” OR AB “blood volume” OR AB “blood donor*”  TI “sport*" OR TI "exercis*" OR TI "physical endurance" OR TI "hiking" OR TI "cycling" OR TI "athletic" OR TI "training" OR TI "working out" OR TI "work out" OR TI "strenuous activity" OR AB “sport*" OR AB "exercis*" OR AB "performance" OR AB "physical endurance" OR AB "hiking" OR AB "cycling" OR AB "athletic" OR AB "training" OR AB "working out" OR AB "work out" OR AB "strenuous activity" OR TI “VO2max” OR TI “maximal aerobic power” OR TI “Oxygen carrying capacity” OR TI “VO2 max” OR AB “VO2max” OR AB “maximal aerobic power” OR AB “Oxygen carrying capacity” OR AB “VO2 max”  TI “men” OR TI “man” OR TI “male” OR TI “sportsman” OR TI “sportsmen” OR TI “woman” OR TI “women” OR TI “female” OR TI “sportswomen” OR TI “Sportswoman” OR TI “athlete” OR TI “adult” OR TI “healthy” OR TI “humans” OR TI “people” OR TI “fit” OR AB “men” OR AB “man” OR AB “male” OR AB “sportsman” OR AB “sportsmen” OR AB “woman” OR AB “women” OR AB “female” OR AB “sportswomen” OR AB “Sportswoman” OR AB “athlete” OR AB “adult” OR AB “healthy” OR AB “humans” OR AB “people” OR AB “fit” |
