# Supplementary material for: The acute effects of whole blood donation on cardiorespiratory and haematological factors in exercise: A systematic review
Source: PLoS One. 2019 Apr 16;14(4):e0215346. doi: 10.1371/journal.pone.0215346 (PMC6467450; doi:10.1371/journal.pone.0215346)
Supplement: S2 Table — (DOCX) [file pone.0215346.s002.docx]

Table 1: Inclusion criteria for study selection

| **PICO Component** | **Inclusion criteria** |
| --- | --- |
| Population | Healthy adults (pathology free), Male and female, 17 - 66 years.  Cardio-vascular exercise |
| Interventions | Whole blood donation (post/Bled) of 470 ml (UK guidelines) followed by aerobic exercise 24 – 48 hr post donation.  Also donations from 400 ml up to 500 ml. |
| Comparators | Aerobic exercise performed with no blood donation (pre/unbled). |
| Outcomes | Haematological variables such as RBC’s, Hb and Hct  Aerobic and cardiorespiratory/respiratory indices including HR, V̇O_2_, V̇O_2max_, V̇O_2peak_, time to exhaustion, watts and exercise capacity. |
