# Supplementary material for: The acute effects of whole blood donation on cardiorespiratory and haematological factors in exercise: A systematic review
Source: PLoS One. 2019 Apr 16;14(4):e0215346. doi: 10.1371/journal.pone.0215346 (PMC6467450; doi:10.1371/journal.pone.0215346)
Supplement: S3 Table — (DOCX) [file pone.0215346.s003.docx]

Variables extracted from research to form data set for analysis.

| **Variable** | **Unit** | **Acronym** |
| --- | --- | --- |
| Haemoglobin | g^.^dL^-1^ | Hb |
| Haematocrit | % | Hct |
| Erythrocytes | Mio μL^-1^ | RBC’s |
| Maximal oxygen uptake | ml∙kg^-1^∙min^-1^ & l∙min^-1^ | V̇O_2max_ |
| Oxygen uptake | ml∙kg^-1^∙min^-1^ & l∙min^-1^ | V̇O_2_ |
| Maximum heart rate | b∙min^-1^ | HR_max_ |
| Maximum aerobic power output | W & W∙kg^-1^ | W_max_ |
| Time/duration to exhaustion | s | TEx |
