# Supplementary material for: The acute effects of whole blood donation on cardiorespiratory and haematological factors in exercise: A systematic review
Source: PLoS One. 2019 Apr 16;14(4):e0215346. doi: 10.1371/journal.pone.0215346 (PMC6467450; doi:10.1371/journal.pone.0215346)
Supplement: S4 Table — (DOCX) [file pone.0215346.s004.docx]

Table 1: Based on the COSMIN doctrine, studies should score an average of >3 (ranked as good) to be considered for inclusion, with 9 studies shown to be eligible using the reviewers average scores and the average combined scores of the reviewers. The only exception to this is the Duda et al., (2003) study, where it was felt after discussion between the reviewers that the lead reviewers score should be accepted.

| **Study** | **Mean score from reviewer 1** | **Mean score from reviewer 2** | **Mean combined scores of reviewers** | **Eligible for review (Yes/No)** | **A selection of indicative exclusion reasons** |
| --- | --- | --- | --- | --- | --- |
| Balke, et al., 1954 | 2.53 | 2.47 | 2.50 | No | No power calculation or sample size justification. Lack of sample description. Clinical significance not obvious. Validity & reliability of instruments not addressed. |
| Bennet & McKay, 2012 | 2.17 | 2 | 2.08 | No | No power calculation or sample size justification. Research design is poorly justified, not stated or inaccurate. Findings extend beyond data set & don’t reflect aims. Clinical significance is over stated. Validity & reliability of instruments not addressed. |
| Brinbaum, Dahl & Boone., 2006 | 2.82 | 2.88 | 2.85 | No | No power calculation or sample size justification. Internal issue of validity from research design. Clinical significance not obvious or over stated. |
| Burnley, et al., 2006 | 3.41 | 2.94 | 3.18 | Yes | N/A |
| Duda, et al., 2003 | 3.00 | 2.88 | 2.94 | Yes | No power calculation or sample size justification. Internal issue of validity from research design. |
| Foster, et al., 2008 | 2.47 | 2.71 | 2.59 | No | No power calculation. Lack of sample description. Research design is poorly justified, not stated or inaccurate. Findings extend beyond data set & don’t reflect aims. Validity & reliability of instruments not addressed. |
| Gordon, et al., 2010 | 3.65 | 3.53 | 3.59 | Yes | N/A |
| Gordon, et al., 2013 | 3.41 | 3.29 | 3.35 | Yes | N/A |
| Hill, Vingren & Burdette., 2013 | 3.35 | 3.18 | 3.26 | Yes | N/A |
| Janetzko, et al., 1998 | 2.65 | 2.65 | 2.65 | No | No power calculation. Research design is poorly justified, not stated or inaccurate. Findings extend beyond data set & don’t reflect aims. Clinical significance not obvious. Validity & reliability of instruments not addressed. |
| Judd, et al., 2011 | 3.24 | 3.06 | 3.15 | Yes | N/A |
| Krip, et al., 1997 | 3.06 | 3.29 | 3.18 | Yes | N/A |
| Markiewicz, et al., 1981 | 2.88 | 2.71 | 2.79 | No | No power calculation or sample size justification. Research design is poorly justified, not stated or inaccurate. Findings extend beyond data set & don’t reflect aims. Clinical significance not obvious. Validity & reliability of instruments not addressed. |
| Meurrens, et al., 2016 | 3.50 | 3.44 | 3.47 | Yes | N/A |
| Mora-Rodriguez, et al., 2014 | 3.06 | 2.76 | 2.91 | No | No power calculation or sample size justification. Internal issue of validity from research design. Some findings lack supporting data set & don’t reflect aims. Clinical significance not obvious. Validity & reliability of instruments not addressed. |
| Panebianco, et al., 1995 | 2.29 | 2.00 | 2.15 | No | No power calculation or sample size justification. Sample description lacks detail, not easily addressing the aim. Research design is poorly justified, not stated or inaccurate. Clinical significance is over stated. Validity & reliability of instruments not addressed. |
| Sliwowski, et al., 2001 | 2.35 | 2.18 | 2.26 | No | No power calculation or sample size justification. Lack of sample description. Research design is poorly justified, not stated or inaccurate. Findings extend beyond data set & don’t reflect aims. Clinical significance not obvious. Validity & reliability of instruments not addressed. |
| Strandenes, et al., 2013 | 2.24 | 2.29 | 2.26 | No | No power calculation or sample size justification. Research design is poorly justified, not stated or inaccurate. Findings extend beyond data set & don’t reflect aims. Clinical significance is over stated. Validity & reliability of instruments not addressed. |
| Williams, Lindhjem & Schuster., 1978 | 2.82 | 2.88 | 2.85 | No | No power calculation or sample size justification. Sample description lacks detail, not easily addressing the aim. Research design is poorly justified, not stated or inaccurate. Clinical significance is over stated. Validity & reliability of instruments not addressed. |
| Ziegler, et al., 2014 | 3.35 | 2.94 | 3.15 | Yes | N/A |
